# The Use of Proton Pump Inhibitors Among Adults in Norway—A Nationwide Drug Utilization Study

**DOI:** 10.1002/prp2.70182

**Published:** 2025-09-30

**Authors:** Reidar Fossmark, Sveinung Molnes, Liv Sagatun, Øyvind Salvesen, Olav Spigset

**Affiliations:** ^1^ Department of Gastroenterology Oslo University Hospital Oslo Norway; ^2^ Department of Clinical and Molecular Medicine Faculty of Medicine and Health Sciences, Norwegian University of Science and Technology Trondheim Norway; ^3^ Department of Gastroenterology and Hepatology St. Olav's University Hospital Trondheim Norway; ^4^ Unit of Applied Clinical Research, Department of Public Health and Nursing, Faculty of Medicine and Health Science Norwegian University of Science and Technology Trondheim Norway; ^5^ Department of Clinical Pharmacology St. Olav's University Hospital Trondheim Norway

**Keywords:** acid inhibition, gastroesophageal reflux disease, pharmacoepidemiology, proton pump inhibition

## Abstract

The use of proton pump inhibitors (PPIs) has increased in Western countries over several decades, and there is concern about unsubstantiated indications and effects of long‐term use. The study aimed to describe the prevalence, incidence, indications, and prescription pattern for PPIs over the past decade in an entire national cohort. This nationwide drug utilization study used data from the Norwegian Prescription Database and Norwegian Patient Registry. Patterns of PPI use were investigated from 2009 to 2022. Prevalent, long‐term, and incident PPI use were calculated. The indication for PPI was defined by the diagnosis code linked to each prescription. Upper endoscopies during the study period were assessed for incident PPI users. PPI prescription increased from 58.1 mill DDD in 2009 to 166.9 mill DDD in 2022, that is, 43 and 106 DDD/1000 inhabitants/day. Prevalent PPI use increased from 3.5% to 9.9%, paralleling an increased incidence from 2.9% to 5.4%. The dominant indications in 2009 and 2022 were esophageal disease in primary care (72.5% and 57.6% of total DDDs) and GERD in specialist care (10.7% and 4.7% of total DDDs). Musculoskeletal disorders, pain, and ulcer prophylaxis all increased during the period. Upper endoscopy around incident PPI use decreased from 25.3% to 11.1%. In conclusion, the 2.46‐fold increase in prevalent PPI use was caused by an absolute increase in PPI prescribed against esophageal disease and GERD, and prophylaxis in patients using ulcerogenic comedication, mainly prescribed in primary care. The findings may help inform strategies to reduce a probable overuse of PPIs.

## Graphical abstract

A nationwide study covering the years 2009–2022 explored trends in proton pump inhibitor use and possible explanations for their increase.

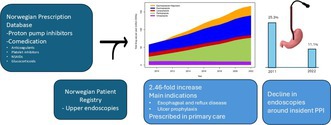

**Abbreviations**
ATC: anatomical therapeutic chemical
GERD: gastroesophageal reflux disease
GI: gastrointestinal
H2RA: histamine‐2 receptor antagonist
ICD: International Classification of Diseases
ICPC: International Classification of Primary Care
NDMA: N‐nitrosodimethylamine
NNT: number needed to treat
NOMESCO: Nordic Medico‐Statistical Committee
NorPD: Norwegian Prescription Database
NPR: Norwegian Patient Registry
NSAID: nonsteroidal anti‐inflammatory drug
PPI: proton pump inhibitor
RAHS: rebound acid hypersecretion

## 1 Introduction

The use of proton pump inhibitors (PPIs) has increased over the last decades in many Western countries. A Danish study found that the prevalence of PPI use increased fourfold from 2002 to 2014 [1]. A similar study from Iceland described a twofold increase from 2003 to 2015, reaching an annual prevalence rate of 15.5% [2]. Annual prescription rates of 10.5%–14.4% have recently been reported in Sweden [3]. Pharmacoepidemiologic studies have utilized data with differing levels of granularity, and further research may enhance the understanding of the factors contributing to the increased use of PPIs.

One of the main indications for long‐term use of PPI is gastroesophageal reflux symptoms or disease. PPIs are also used as prophylaxis against gastroduodenal ulcerations in patients using nonsteroidal anti‐inflammatory drugs (NSAIDs) as well as acetylsalicylic acid, and there are recommendations that patients with increased risk of upper gastrointestinal bleeding should use such prophylaxis [4].

Dyspepsia is a highly prevalent symptom in the general population and the global prevalence has been estimated to be about 20% [5, 6]. Considering that previous and current guidelines recommend PPI treatment for individuals with functional or uninvestigated dyspepsia, this may contribute to an increase in PPI prescriptions [7, 8]. Even after stopping short‐term PPI use, a high proportion of individuals develop symptoms due to rebound acid hypersecretion [9, 10, 11, 12, 13] and a significant proportion of patients initially prescribed short‐term treatment eventually become long‐term users [14]. These factors could contribute to the observed increase in long‐term use of PPIs.

There is an extensive literature that describes the potential adverse effects of PPI use, which include nutrient deficiencies, increased fracture risk, renal disease, respiratory tract and bile duct infections, neurological diseases, liver cancer, and gastric cancer [15, 16]. Furthermore, numerous studies in various settings have found that the prescription of PPIs is often inappropriate [17], particularly in the elderly. A combined rapid increase in inappropriate use of PPIs is of particular concern both from an individual risk/benefit perspective and related to costs at the population level. A national study from France explored PPI use in 2015 and found that 32% of patients did not have a diagnosis supporting PPI prescription [18].

Aggregated data of PPI prescriptions found that the proportion of the population in Norway that received a prescription of a PPI increased from 6.5% to 10.8% during the time period 2011–2020 [19] and we wanted to further explore possible explanations for this increase.

## 2 Materials and Methods

### 2.1 Data Sources

Nationwide data were retrieved from the Norwegian Prescription Database (NorPD) and consisted of information about dispensed prescription drugs. The study population consisted of all adults ≥ 18 years of age with at least one PPI prescription during the study period from January 1, 2009 to December 31, 2022. National data from the Norwegian Patient Registry (NPR) on all upper endoscopies performed during the study period on all individuals in the study population were combined with NorPD data by means of the unique national identity number assigned to every person living in Norway. Upper endoscopies were here identified by the Nordic Medico‐Statistical Committee (NOMESCO) procedure codes for the study period (Upper endoscopy without biopsy JUD02 and UJD02, upper endoscopy with biopsy JUD05 and UJD05).

### 2.2 Study Drugs and Definitions

PPIs were defined by their Anatomical Therapeutic Chemical (ATC) codes A02BC01–C05 (omeprazole, pantoprazole, lansoprazole, rabeprazole, and esomeprazole) in addition to M01AE52, which comprises an esomeprazole and naproxen combination product marketed in Norway. Rabeprazole has not been prescribed in Norway in the study period. Concurrent use of selected comedications was assessed for the following drugs defined by their ATC codes B01AA, B01AB, B01AE, B01AF, and B01AX (anticoagulants), B01AC (platelet inhibitors), M01AB, M01AC, M01AE, and M01AH (NSAIDs), and H02AB (corticosteroids for systemic use). Doses were expressed in defined daily doses (DDDs) as listed in the ATC/DDD Index published by the WHO Collaborating Centre for Drug Statistics Methodology [20].

The overall PPI use in Norway is presented as the number of dispensed DDDs to the adult population for each year. To enhance readability, we generally use the word “prescription” in the further text even though the included data represent drug dispensing at the pharmacies.

Prevalent PPI use was calculated from the number of PPI users on a day‐by‐day basis throughout the study period. A subject was defined as a PPI user for the days included in the period covered by the DDDs [20] available from each prescription, assuming a consumption of one DDD per day, and was therefore calculated from January 1, 2010. Long‐term PPI use was defined as prescription of at least 180 DDDs for at least the two previous years. This variable could thus by definition be calculated from January 1, 2011. The adult population of Norway was estimated as of July 1st for each year, and this number was used as the denominator in the prevalence calculations and for calculation of prescription per 1000 inhabitants [21]. This variable was available for the total population and various age groups as well as for males and females separately.

Incident PPI use was defined as a PPI prescription without any PPI prescription for at least the two previous years and could therefore be calculated from January 1, 2011. It was also assessed whether incident PPI users underwent an upper endoscopy 180 days before or after incident PPI use.

The indication for PPI prescriptions was assessed by the use of International Classification of Primary Care (ICPC) 2nd version or the International Classification of Diseases (ICD‐10) codes for prescriptions, which are the coding systems used by primary care and specialist health care, respectively (Table S1). Prescriptions of drugs where expenses are reimbursed by the national health service have a diagnosis linked to each prescription, and the classification system (ICPC or ICD‐10) was also used to categorize prescriptions to come from primary care or secondary health care. Information about the prescribing physician's specialty was also provided for each prescription. However, as a physician may have two or multiple specialties, the subspecialty assessed to be most relevant for PPI prescription was then recorded (e.g., general practice was recorded for doctors with additional specialties; internal medicine was recorded if not also a gastroenterologist or nephrologist who were also specialists in internal medicine).

Comedication was defined as prescription of the predefined drugs listed above, within 90 days leading up to a PPI prescription as well as all concurrent use defined as the period covered by a PPI and a comedication based on the DDD prescribed and assumed use of one DDD per day for all medications. Due to the 90‐day time window, this variable was calculated from January 1, 2010.

### 2.3 Analyses

Analyses were performed using mySQL version 8.0.42 and R version 4.3.3. GraphPad Prism 10 (GraphPad Software, Boston, MA, USA) was used for the generation of figures presenting aggregated data.

### 2.4 Ethics

Ethical approval was obtained from the Regional committees for medical and health research ethics in Central Norway (reference number 470404). All information was de‐identified prior to analyses, and studies based on national registry data do not, according to Norwegian legislation, require informed consent from individuals.

## 3 Results

The key results are presented in Table 1.

**TABLE 1:** Key variables illustrating PPI prescription in Norway in 2009 and 2022.

	2009	2022	Percent change
Total prescription (DDD/1000 inhabitants/day)	43.0	106.0	+146
Prescribed DDDs to females (%)	52.2	54.7	+4.8
Prescribed DDDs to subjects > 60 years of age (%)	58.5	58.6	+0.2
Upper endoscopy around incident PPI use (%)	25.3	11.1	−56.1
Prescribed DDDs (millions) and diagnosis			
Esophageal disease/GERD	48.3	104.0	+215
Ulcer prophylaxis, musculoskeletal disease and pain^a^	0.66	22.5	+3309
Prescription in general practice (%)	77.8	75.2	−3.3
Non‐reimbursed prescriptions (%)	10.2	5.2	−49.0

Abbreviations: DDD, defined daily dose; GERD, gastroesophageal reflux disorder.

^a^ Includes PPI as prophylaxis of drug‐induced stomach and duodenal ulcers and all use of a naproxen and esomeprazole combination product.

### 3.1 Total PPI Use

**FIGURE 1:**
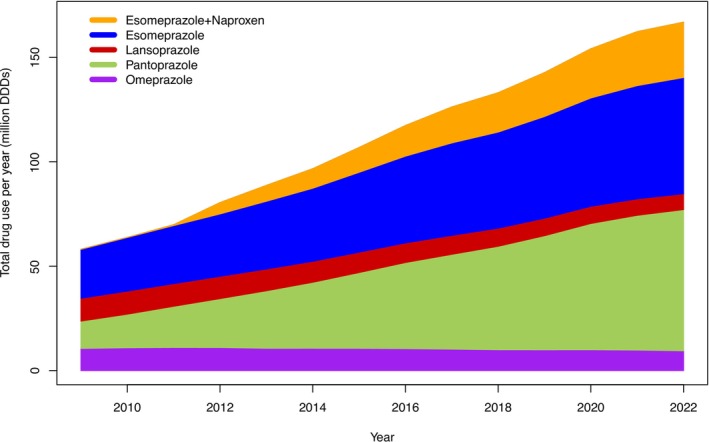
The total number of prescribed DDDs of PPIs for each year (in millions) in adults in Norway from 2009 to 2022.

The use of PPIs increased from 58.1 mill DDD in 2009 to 166.9 mill DDD in 2022, and the absolute increase in DDDs was driven by a 3.5‐fold increase in esomeprazole and a fivefold increase in the use of pantoprazole. The adult population in Norway increased from 3 695 771 to 4 316 747 individuals over the study period, and the PPI use adjusted for population size increased from 43.0 DDD/1000 inhabitants per day in 2009 to 106.0 DDD/1000 inhabitants per day in 2022. This increase in DDDs was observed in particular for pantoprazole and for esomeprazole alone or in combination with naproxen (Figure 1).

**FIGURE 2:**
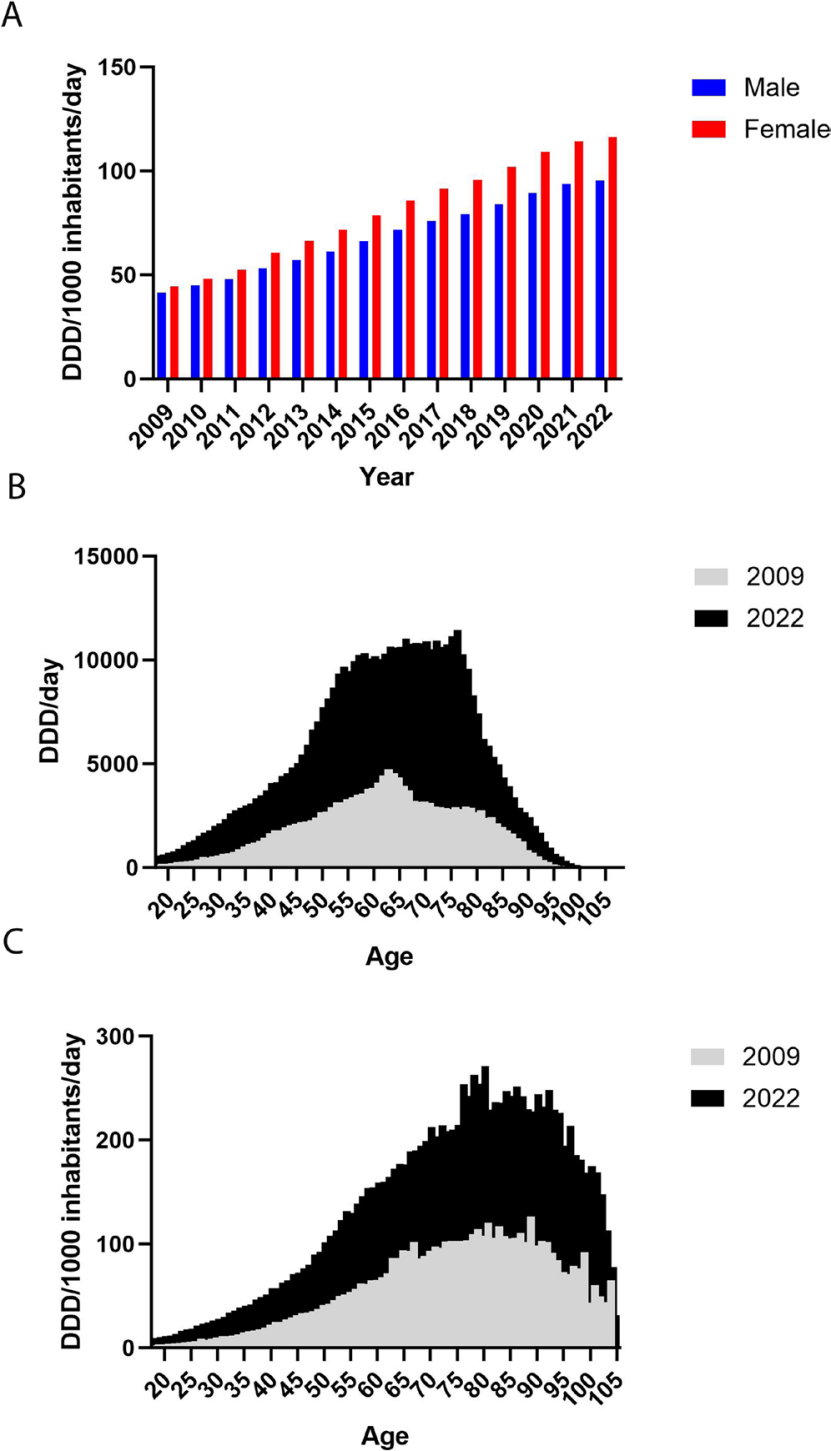
Female patients used more PPI than male patients during the entire study period, and the difference increased with time (A). The age distribution was similar in 2009 and 2022, overall (B) and when adjusting for population size (C).

The population‐adjusted use of PPIs increased during the period 2009 to 2022 from 42.0 to 106.0 DDD/1000 inhabitants/day, with a similar increase in both the male and female population (Figure 2). The increase in PPI use was evident in all age groups (Figure 2). The higher total DDD prescribed to female inhabitants was evident in all age groups from 18 to 90 years (Figure S1). Patients aged 60 years and above accounted for the majority of the total DDDs, with a stable distribution observed throughout the study period. Specifically, individuals > 60 years of age dispensed 58.5% of total DDDs in 2009 and 58.6% in 2022.

Sensitivity analyses conducted across Norway's four health regions revealed that, while PPI use increased uniformly, the overall national trend was not attributable to divergent regional prescribing patterns (Figure S2).

### 3.2 Prevalence, Incidence and Indications

**FIGURE 3:**
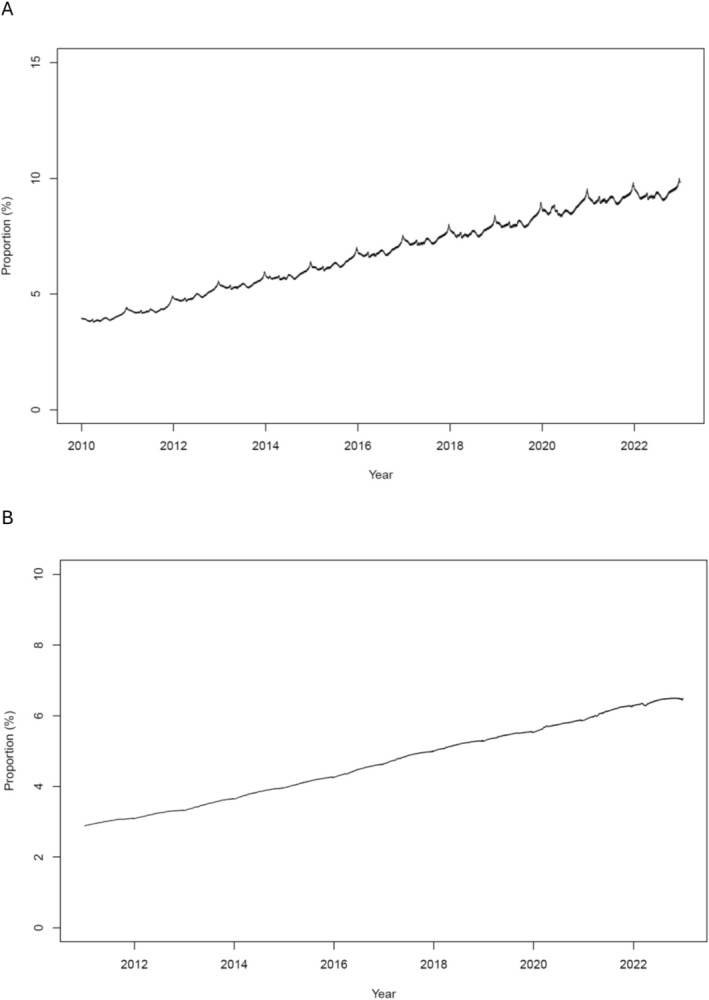
The proportions of the Norwegian adult population that were prevalent PPI users from January 1, 2010 to December 31, 2022 (A) and prevalent long‐term PPI users from January 1, 2011 to December 31, 2022 (B).

The proportion of the population who were prevalent PPI users increased during the study period from 3.5% in 2010 to 9.9% in 2022 (Figure 3A). Similarly, the proportion of the population that were long‐term users also increased, from approximately 3% to 6% (Figure 3B). Incident PPI use increased from 2.9% of the adult population in 2011 to 5.4% in 2022.

There were 2.27 mill cases of incident PPI use in the entire study period, and the proportion of PPI users that underwent upper endoscopy around treatment start decreased from 25.3% in 2011 to 11.1% in 2022. In patients with esophageal disease (ICPC diagnosis) or GERD (ICD‐10 diagnosis) combined, the proportion examined by upper endoscopy around treatment start fell from 41.4% to 27.1%, while in PPI users without a reimbursement diagnosis, the proportion fell from 19.2% to 6.9%.

**FIGURE 4:**
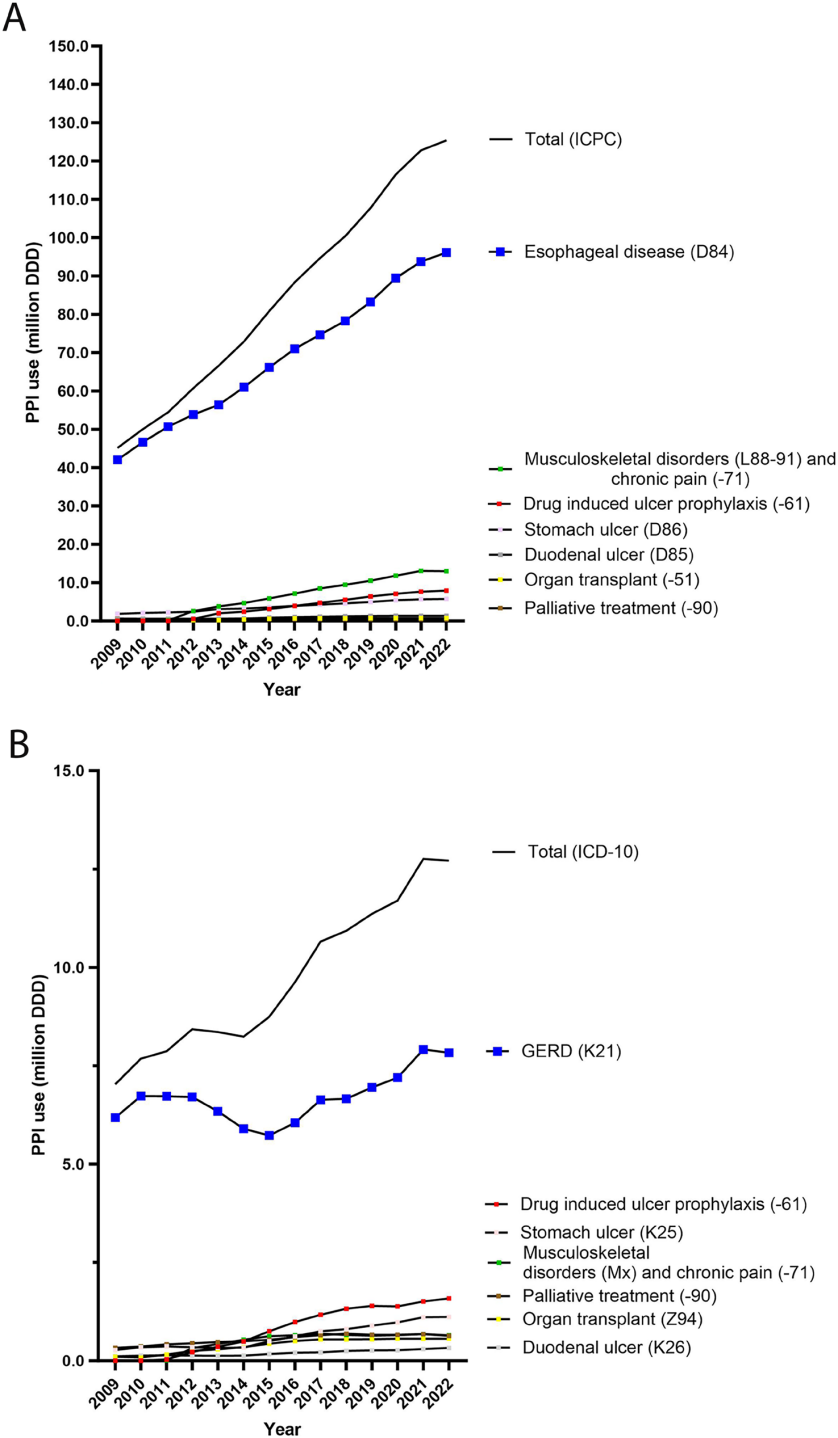
PPI prescription for the most common diagnoses in primary care (ICPC diagnoses) (A) and specialist care (ICD‐10 diagnoses) (B).

Esophageal disorders and GERD were the predominant indications for PPIs throughout the study period. While the increase in prescriptions in specialist health care was modest, the prescriptions in primary care more than doubled. In 2009, the dominant indications in primary and specialist health care, respectively, were esophageal disease and GERD (72.5% and 10.7% of DDDs overall), as compared to gastric ulcer (3.3% and 0.46% of DDDs overall) and duodenal ulcer (0.86% and 0.21% of DDDs overall) (Figure 4). In 2022, esophageal disease and GERD in primary and specialist care respectively were still dominant indications, but their proportions declined in 2022 compared to 2009 (57.6% and 4.7% of the DDDs overall). Furthermore, musculoskeletal disorders and pain (7.1% and 0.39% of DDDs overall, including the combined esomeprazole and naproxen product) and prophylaxis against gastroduodenal ulcer (4.8% and 0.95% DDDs) were more common indications. It should be noted that the combined esomeprazole and naproxen product was reimbursed from 2011, and musculoskeletal disorders (ICPC and ICD‐10) appeared among the dominant indications. The proportion of DDDs overall which were prescribed in primary care was 77.8% (45.2 of 58.1 million DDDs) in 2009 and 75.2% (125.4 of 166.9 million DDDs) in 2022. Prescriptions with no (reimbursement) diagnosis constituted 10.2% of DDDs in 2009, compared to 5.2% of the DDDs in 2022, and the proportions prescribed in primary versus specialist health care could not be determined for these prescriptions.

### 3.3 Comedication

**FIGURE 5:**
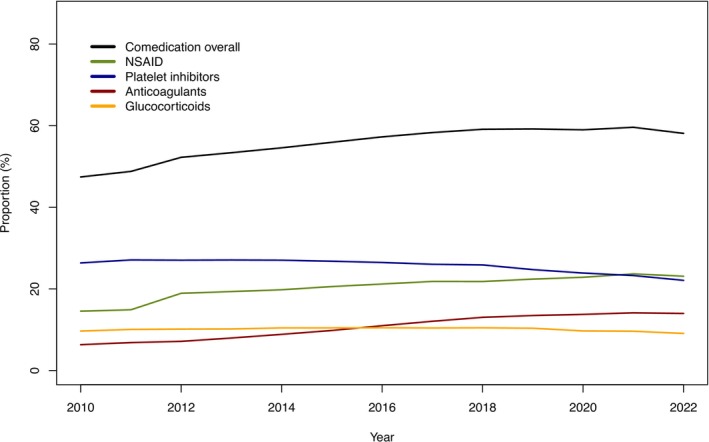
Proportions of PPI users with main classes of comedication.

The use of predefined comedications was high during the entire study period (Figure 5). The most prevalent class of comedication was platelet inhibitors; however, the proportion that used this comedication declined slightly with time. Comedication with NSAIDs gradually increased from 2011 to become the most common class in 2022. The use of systemic glucocorticoids in PPI users was fairly constant during the study period, while comedication with anticoagulants increased.

### 3.4 Prescribing Physician Specialty

**FIGURE 6:**
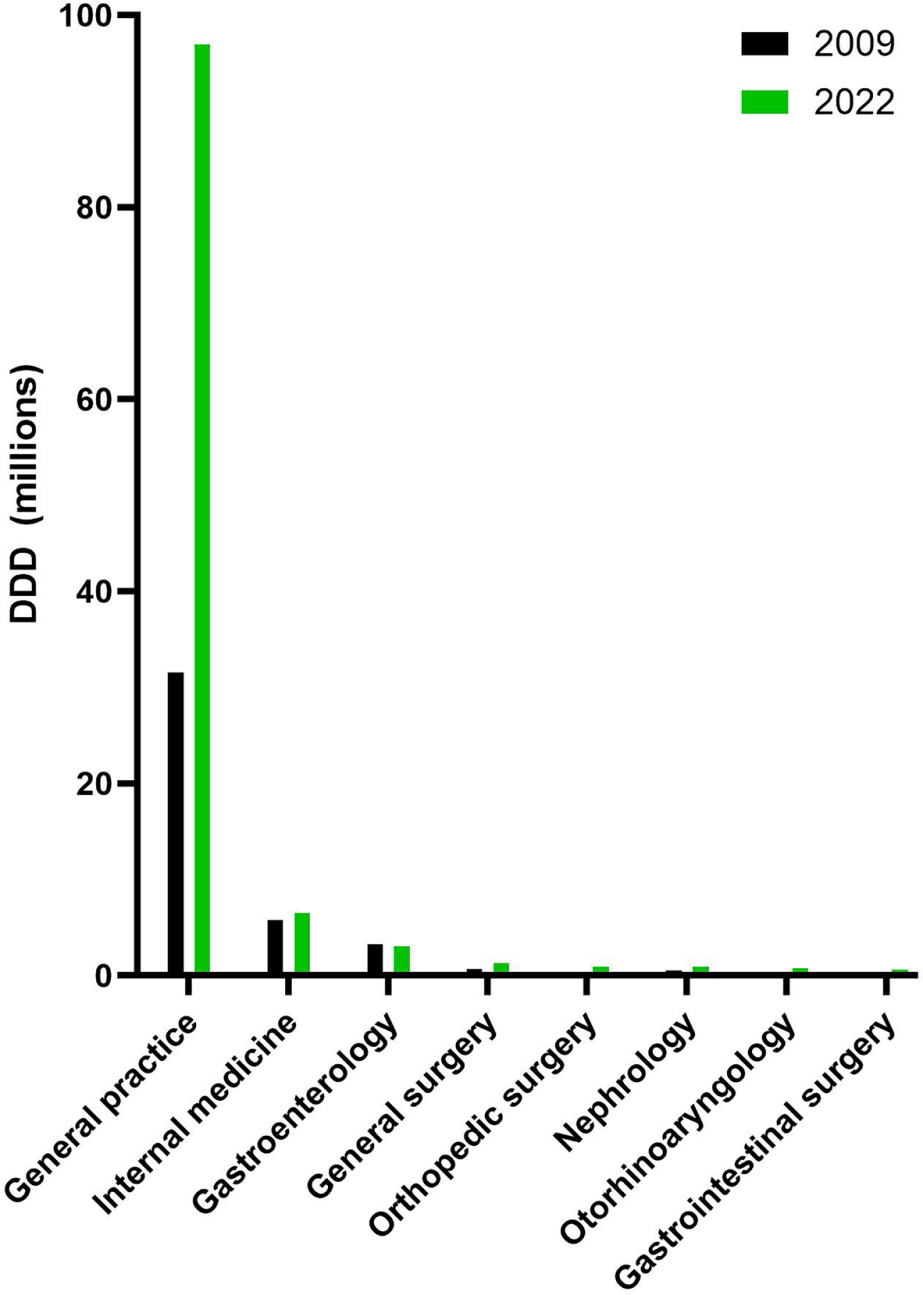
Top eight specialties of the physicians prescribing PPIs in 2009 and 2022. Those with an internal medicine subspecialty not being among those depicted (i.e., all other than gastroenterology and nephrology) are listed as “Internal medicine.”

The specialties of the prescribing physicians are presented in Figure 6. Of those with a registered specialty, general practitioners prescribed the majority of PPIs in 2009 (74.1%), which increased until 2022 (87.4%). All other specialists, including specialists in internal medicine or gastroenterology, only prescribed minor or minimal proportions of the total DDDs.

## 4 Discussion

The marked increase in PPI use in Norway over recent decades prompted an investigation to explore potential underlying explanatory mechanisms. Both prevalent and incident PPI use increased gradually during the study period. The largest single fraction of the total DDDs was prescribed for esophageal disease in general practice, constituting more than half of the total number of DDDs. Prophylactic use of PPIs in patients using drugs which may increase the risk of ulcerations in the upper GI tract also increased. The proportion of patients who underwent upper endoscopy around initiating PPI treatment fell for the population overall and specifically in patients with esophageal disease or GERD.

The strengths of this study included complete national coverage of all prescriptions that were dispensed. The study also had information about DDDs dispensed for each prescription and patient, the diagnosis linked to each prescription indicating the patient setting, as well as information about the prescribing physicians' specialty at the time of prescription. The study also had access to information about indication, as well as comedication on an individual patient level, so that reliable prevalence and incidence rates could be calculated. PPIs became available over the counter in Norway during the study period, and this use could not be estimated and is also not included in the study; however, only small packages of pantoprazole 20 mg are sold, and the access to prescriptions with reimbursement of drug expenses was facilitated during the study period. The study was limited by the lack of information about PPI use in patients admitted to hospitals and nursing homes. Although we use the term “prescriptions” in this article, the actual number of prescriptions is higher, as the variables registered are related to drugs dispensed from pharmacies. A nationwide Norwegian study from 2016 to 2018 found that 13% of all prescriptions of pantoprazole were never dispensed [22]. It is reasonable to assume that the situation is the same for other PPIs. On the other hand, an unknown proportion of the drugs dispensed are not ingested by the patients. Therefore, the results presented here do neither represent the number of PPIs prescribed nor the amount taken by the patients. Finally, the diagnosis linked to each prescription may also have been adapted to reimbursable diagnoses. However, given that suspected esophageal disease was approved for reimbursement, this limitation should not affect the main findings.

Throughout the study period, the main indications for PPI use were esophageal disease and GERD. Symptoms of gastroesophageal reflux are common in the general population, and the number of individuals consulting a physician with such complaints may have increased during the study period. It is well‐documented that increasing body mass index (BMI) is a risk factor for such symptoms [23]. Although overweight and obesity have increased in Norway during the study period [24], this is unlikely to explain the magnitude of increased PPI use. The requirement of a confirmed diagnosis of GERD by upper endoscopy or 24 h esophageal pH monitoring for reimbursement was removed in 2014, which may have led to a slight increase in prescriptions. Long‐term PPIs may then more often have been initiated by primary care physicians.

A high and constant proportion (75%–77%) of the DDDs was prescribed in primary care. The large majority of incident PPI users did not undergo upper endoscopy around PPI initiation, and the proportion of patients who were examined fell during the study period. Several factors could contribute to this practice. Clinical guidelines recommend the use of PPIs for patients with functional dyspepsia [25], even though the number needed to treat (NNT) is high and the placebo response does by far exceed the actual therapeutic effect of PPIs [26]. Similarly, an 8‐week trial of PPI for patients with symptoms of GERD has been recommended in several guidelines [27]. This practice is problematic considering that rebound acid hypersecretion (RAHS) following PPI discontinuation is a well‐documented phenomenon [9, 11, 12, 13]. RAHS can lead to acid‐related symptoms in up to half of patients after just 4 weeks of PPI use, which may cause continued long‐term use once treatment has started.

Histamine‐2 receptor antagonists (H2RAs) represent a viable alternative to PPIs due to their rapid onset of action [28, 29], and H2RAs are therefore particularly suitable for patients with mild and intermittent symptoms from the upper gastrointestinal tract [26]. The use of H2RAs in Norway decreased from 7.3 million DDDs in 2011 to 0.15 million DDDs in 2020 [19] as studies published in 2019 found the probably carcinogenic substance N‐nitrosodimethylamine (NDMA) as a contaminant in ranitidine products, and all preparations containing ranitidine were recalled from the market [30]. The use of other H2RAs in Norway, such as famotidine, is currently limited by the requirement for individual reimbursement applications. Consequently, prescribing PPIs is a more convenient option for physicians managing mild and intermittent upper abdominal symptoms.

The more recent indications of PPI use included gastroprotection in patients using platelet inhibitors or having musculoskeletal disease as reimbursement diagnoses. The concurrent use of NSAIDs and PPIs became apparent during the study period. Concomitant use of anticoagulants and platelet inhibitors has also increased in Norway [19], and international guidelines published during the study period recommend prophylactic use of PPIs to reduce the risk of upper gastrointestinal (GI) bleeding in individuals with high risk of an event [31]. Age over 60 years is considered one such risk factor [31] and the majority of PPI users in Norway were > 60 years. The protective effect of PPI in patients with combined use of acetylsalicylic acid and another anticoagulant was evaluated in a randomized controlled trial with 17 598 participants included [32]. There were no significant differences in the primary outcome, which was a clinically significant upper GI event, and the NNT was 1770 over 3 years [33]. The study illustrates that prophylaxis could be clinically relevant only in patients with higher risk of an event than the study population. It has also been proposed that there may be overuse of gastroprotection with PPIs in NSAID users, as a large meta‐analysis found that the NNT to prevent one GI bleeding was approximately 100 [34]. In populations with lower absolute risk, clinicians may overestimate the potential benefit of risk reduction, since gastroprotection with a PPI is generally recommended only for patients with a sufficiently high baseline risk [35]. These combined indications for gastroprotection with PPIs may account for the second largest share of the overall increase.

The concerns over side effects and drug costs are especially relevant if PPIs are prescribed without an approved indication to a large number of patients. The range of possible side effects described in the literature is wide, and many of the proposed side effects found in epidemiological studies are supported by mechanistic and experimental studies. In addition to concerns about nutrient deficiencies, electrolyte disturbances, and gastric cancer, other side effects seem plausible as a consequence of hypoacidity on the defense against microbes [36]. Various side effects of PPIs may be mediated by changes in the gastrointestinal microbiome [37], which has again been associated with the risk of intestinal diseases [38, 39, 40] as well as other disorders [41, 42].

The increased use of PPI has been reported from several Western countries over the past decade [1, 2, 3, 18] and this trend has been observed despite possible differences in national reimbursement policies, in marketing, and national prescription guidelines. An overuse of PPIs is likely and a national campaign funded by the Norwegian Ministry of Health and Care Services was started in 2024 to improve PPI stewardship and reduce unnecessary PPI use [43].

## 5 Conclusions

In this nationwide study, a 2.8‐fold increase in prevalent PPI use was observed during the 14‐year study period from 2009 to 2022. This was caused by a large increase in PPI use against esophageal disease and GERD, as well as prophylaxis in patients receiving ulcerogenic comedication. The proportion of incident PPI use in patients investigated by upper endoscopy was halved. The current findings may help inform strategies to reduce the probable overuse of PPIs at a population level.

## Author Contributions

**Reidar Fossmark:** conceptualization, investigation, methodology, funding acquisition, writing – original draft, writing – review and editing, formal analysis, supervision. **Sveinung Molnes:** investigation, methodology, writing – review and editing. **Liv Sagatun:** conceptualization, methodology, writing – review and editing. **Øyvind Salvesen:** methodology, formal analysis, visualization. **Olav Spigset:** conceptualization, methodology, funding acquisition, writing – review and editing.

## Conflicts of Interest

The authors declare no conflicts of interest.

## Supporting information


**Data S1:** prp270182‐sup‐0001‐DataS1.docx.

## Data Availability

The data can be shared after approval by the Regional committees for medical and health research ethics in Central Norway.
