# Supplementary material for: The Use of Proton Pump Inhibitors Among Adults in Norway—A Nationwide Drug Utilization Study
Source: Pharmacol Res Perspect. 2025 Sep 30;13(5):e70182. doi: 10.1002/prp2.70182 (PMC12483766; doi:10.1002/prp2.70182)
Supplement: Supplementary file 1 — Data S1: prp270182‐sup‐0001‐DataS1.docx. [file PRP2-13-e70182-s001.docx]

**The use of proton pump inhibitors among adults in Norway – a nationwide drug utilization study**

**Supplementary material**

Supplementary Figure 1. The age distribution of PPI prescription in males and females in 2022.


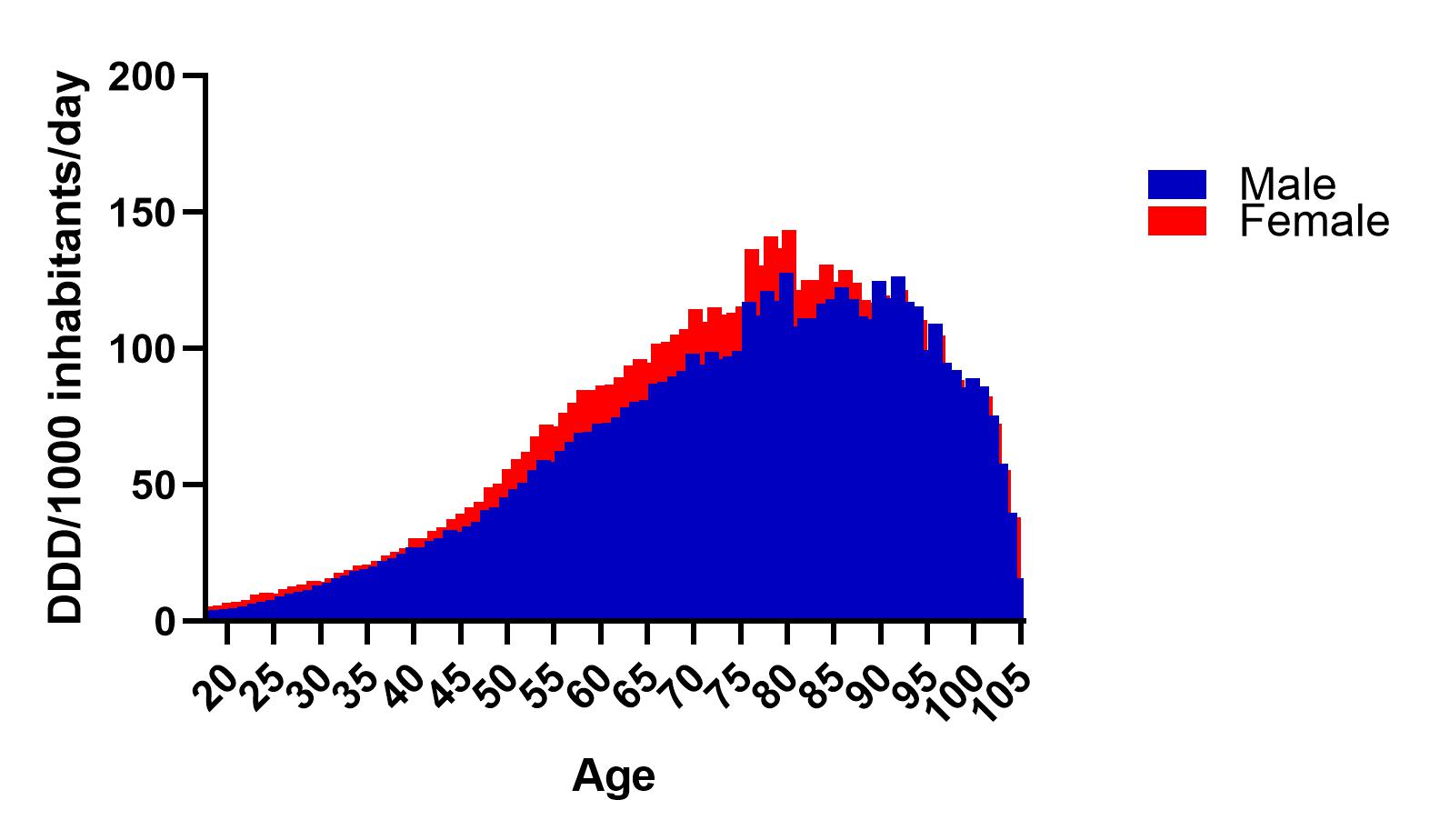


Supplementary Figure 2. Regional differences in PPI use (DDD/1000 inhabitants) annually from 2009 to 2022.


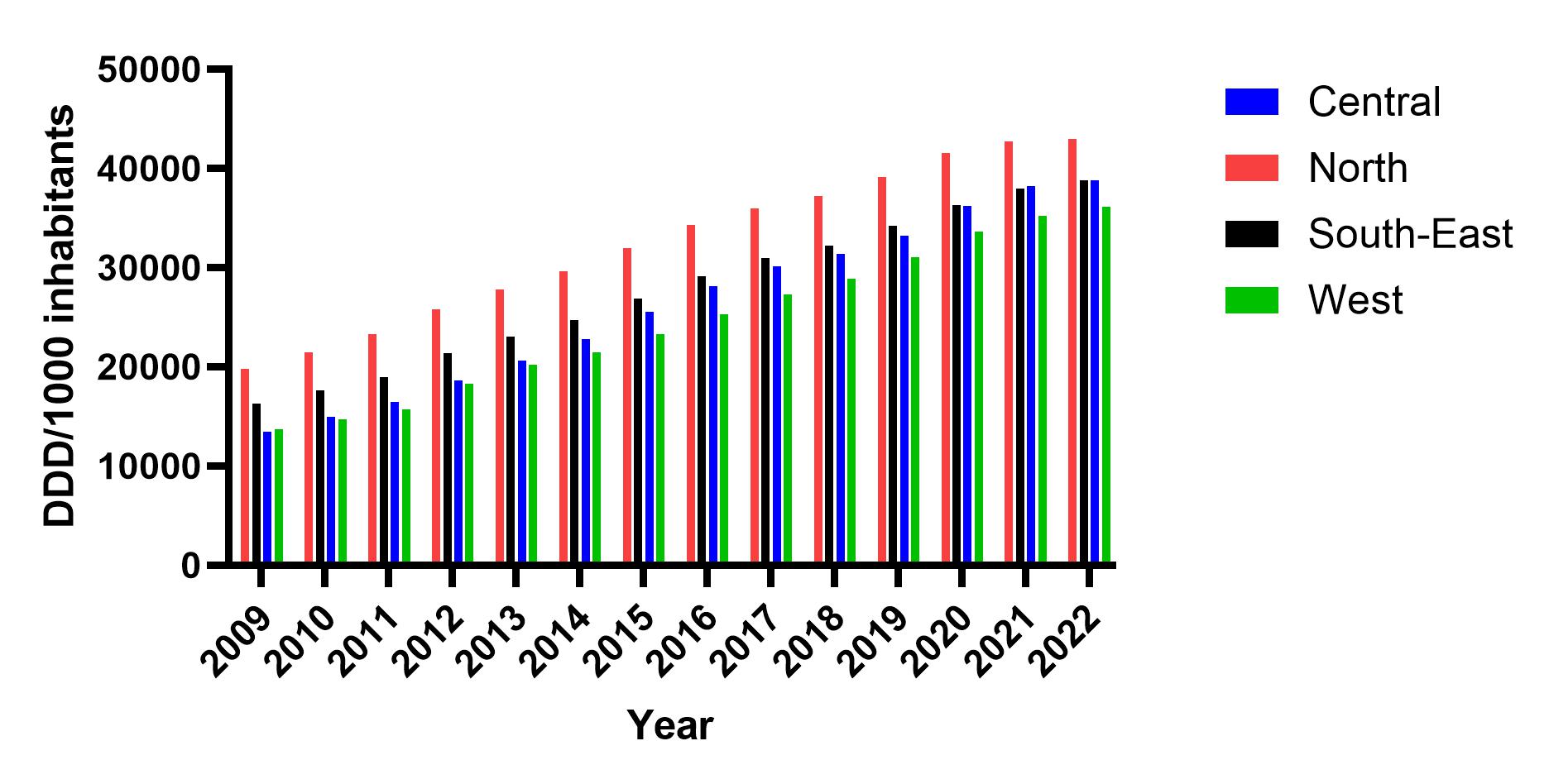


Supplementary Table 1. The most prevalent ICD-10 and ICPC codes used as reimbursement diagnoses for PPIs.

| ICPC |  |  |
| --- | --- | --- |
|  | D84 | Esophageal disease |
|  | L88 | Rheumatoid arthritis / rheumatic disease |
|  | L89 | Hip arthrosis |
|  | L90 | Knee arthrosis |
|  | L91 | Arthrosis |
|  | -71 | Chronic pain |
|  | -61 | Drug induced ulcer prophylaxis |
|  | D86 | Stomach ulcer |
|  | D85 | Duodenal ulcer |
|  | -51 | Organ transplant |
|  | -90 | Palliative treatment |
| ICD-10 |  |  |
|  | K21 | Gastroesophageal reflux disease |
|  | -61 | Drug induced ulcer prophylaxis |
|  | K25 | Stomach ulcer |
|  | K26 | Duodenal ulcer |
|  | Mx | Musculoskeletal disorders |
|  | -71 | Chronic pain |
|  | -90 | Palliative treatment |
|  | Z94 | Organ transplant |

ICPC: International classification of primary care; ICD-10: International classification of diseases and health related problems, 10^th^ edition.
